# The evolution of the health system outcomes in Central and Eastern Europe and their association with social, economic and political factors: an analysis of 25 years of transition

**DOI:** 10.1186/s12913-016-1344-3

**Published:** 2016-03-17

**Authors:** Piotr Romaniuk, Adam R. Szromek

**Affiliations:** Department of Health Policy, School of Public Health in Bytom, Medical University of Silesia in Katowice, ul. Piekarska 18, Bytom, 41-902 Poland; Department of Computer Science and Econometrics, Faculty of Organization and Management, Silesian University of Technology, ul. Roosevelta 26-28, Zabrze, 41-800 Poland

**Keywords:** Health system outcomes, Health reforms, Health system transition, Central and Eastern Europe, Post-communist countries

## Abstract

**Background:**

After the fall of communism, the countries of Central and Eastern Europe started the process of political, economic, and social transformation. In health system the reform directions were often similar, despite differences in transition dynamics and the degree of government determination to implement reforms. Nonetheless, for most post-communist countries, there is a gap in evidence regarding the effectiveness of implemented reforms and their impact on health system performance. The presented study attempts to analyse and evaluate the results of health reforms in CEE countries with regard to their influence on health system outcomes. We also analysed the external and internal health system environments during the transition period to determine the factors affecting the effectiveness of health reforms.

**Methods:**

We compared the indicators of population health status, lifestyle, occupational safety issues and health system resources in 21 post-communist countries between sub-periods across the entire transition period at the aggregate level. The dynamics of change in health system outcomes in individual countries, as well as between countries, was also compared. Finally, we analysed the correlations between health system outcomes gathered into one synthetic measure and factors considered as potential determinants affecting the effectiveness of health reforms. The analyses were performed based on one-dimensional, two-dimensional and multidimensional statistical methods. The data were retrieved from the international databases, such as WHO, World Bank, International Labour Organization, World Value Survey and the European Social Survey.

**Results:**

Among the factors positively stimulating improvements in health system outcomes were the total expenditure on health and a lower financial burden on patients, but primarily they were determined by the broader economic context of the country. Another finding was that better initial position positively determined health system outcomes at later stages, but did not affect the degree of improvements. Countries that embarked on comprehensive reforms early on tended to achieve the greatest improvements in health system outcomes.

**Conclusions:**

Poorer countries may have only limited ability to improve health system outcomes by committing more financial resources to the health system. Progress can still be made in terms of health behaviours, since policies to address these have so far been insufficient or ineffective.

## Background

The defining feature of Central and Eastern European (CEE) countries is their similarity of historical and political experiences prior to 1989. The entire region was part of the Soviet empire, either directly, or as socialist countries adopting the Soviet political model and remaining under Soviet influence. Following the collapse of the communist bloc in 1989 and the dissolution of the Soviet Union in 1991, the countries of the region more or less simultaneously started the process of political, economic, and social transformation. The specifics of approaches adopted during the transformation varied across countries, as did the range and depth of implemented changes. However, the entire group entered broadly similar directions of reform, away from the centrally planned socialist economy and the communist socio-political model, and towards liberal democracy and market economy (although many former Soviet countries failed in the first aspect). We are therefore dealing with a relatively large group of countries that had similar starting points for transition, but often took slightly different tracks (more on this topic: [1–3]).

Quite similar is the situation with regard to the countries’ health systems. Before the collapse of the communist system, countries had applied very similar patterns of organizing their health systems (with a few exceptions, e.g., the former Yugoslavia [4, 5]; more on the similarities between health systems of communist countries: [6]). With the collapse of the communist system, reform directions in the health system were also often similar, despite differences in transition dynamics and the degree of government determination to implement reforms. It is also possible to identify countries where the communist paradigm remained alive, with merely a minimum scope of changes implemented. There are also differences in terms of the reforms being implemented, although these differences often relate only to the reform details, rather than the overall model or paradigm of reform [5], and, obviously, the reform outcome [7].

Existing studies emphasize the commonality of transition features and the generally unprecedented scope of change [7, 8]. Despite this, both the transition course and its foundation raise some doubts. First, the chosen direction of reform used to be often a purely political decision, not backed by clear evidence for the appropriateness of organizational models. Second, for most countries, there is a gap in evidence regarding the effectiveness of implemented reforms and their impact on health system performance [5]. The present study is an attempt to partially fill these gaps about the effects and effectiveness of health reforms in CEE countries by analysing the evolution of their health systems and its results in terms of health system outcomes.

This study had the overall aim to analyse and evaluate the results of health reforms in CEE countries with regard to their influence on population health and health system outcomes. Following on from this overall aim, the following three analyses were undertaken:

1. A comparison of health system outcomes in post-communist countries across different sub-periods of the entire transition period at the aggregate level provided a general picture of how health systems had changed.
2. A comparison of the health system outcomes in individual countries in different sub-periods, as well as the dynamics of their change to determine which of the countries managed to implement the most beneficial package of health reforms.
3. We also analysed the external and internal health system environments during the transition period to determine the factors affecting the evolution of health system outcomes.

## Methods

The study covers a period of 25 years (1988–2012) in 21 CEE countries (Albania, Armenia, Belarus, Bosnia and Herzegovina, Bulgaria, Croatia, Czech Republic, Estonia, Georgia, Hungary, Latvia, Lithuania, Macedonia, Moldova, Poland, Romania, Russia, Serbia, Slovakia, Slovenia, and Ukraine). In some cases, particularly Serbia and Bosnia and Herzegovina, significant gaps in data availability were identified, so that we had to limit the catalogue of countries analysed to 19 in most cases. Each country was characterized using a dataset of the following indicators of population health status: life expectancy, infectious disease mortality, diabetes mortality, cardiovascular disease mortality, infant mortality, maternal mortality, cancer mortality, external cause mortality, and tuberculosis mortality. In addition, countries were assigned values to assess the effectiveness of health policies addressing lifestyle and occupational safety issues: alcohol consumption, the prevalence of regular smokers, tobacco consumption, the number of accidents at work per 1,000 employees, and the number of fatal accidents at work. The third group of indicators referred to health system resources in order to assess how the restructuring processes were run and whether they affected service availability and usage: the number of outpatient visits per person and year, the number of hospital beds per 100,000 population, and the bed occupancy rates in acute care hospitals Individual indicators were taken into account, as well as information on how they changed in subsequent sub-periods of transition.

We then selected factors generally considered as potential determinants of health system outcomes, according to the following four groups (for details, see Table 1) [2, 7]:

- systemic factors,
- economic factors,
- political factors, and
- social factors.

**Table 1:** Factors determining health system outcomes: changes observed in periods A, B, and C

Factors		A	B	C	Mean for 1988–2012
		1988–1996	1996–2004	2005–2012	
	*p*	Mean			
Economic factors					
GDP per capita (PPP, USD)	<0.05	5,789.26	8,065.90	13,589.57	9,680.19
GDP change (%)	<0.05	-1.69	4.58	3.25	2.70
Unemployment	<0.05	9.36	13.75	12.37	12.28
Industry share of GDP	<0.05	38.26	30.78	30.13	32.94
Services share of GDP	<0.05	42.93	57.14	61.54	54.06
Agriculture share of GDP	<0.05	18.72	12. 15	8.36	13.00
Budget deficit (% of GDP)	0.07	-6.00	-2.28	-3.13	-2.90
Gross public debt (% of GDP)	<0.05	33.75	43.10	32.28	36.88
Inflation rate	<0.05	277.01	22.37	6.14	72.79
Total investments (% of GDP)	0.23	25.35	23.79	26.17	25.07
Foreign investments (net millions of dollars in current value)	<0.05	496.87	1,477.40	6,230.11	3,062.03
Foreign investments per capita (dol, current)	<0.05	44.19	151.42	509.49	263.05
Government spending (% of GDP, excluding military)	0.13	18.33	17.97	17.82	18.03
Public expenditure on social policy (% of GDP)	0.93	17.24	16.79	16.69	16.81
Social Factors					
Number of people living below poverty line (national threshold)	0.15	38.60	23.43	19.39	21.71
Human Development Index	<0.05	0.70	0.74	0.78	0.77
Public expenditure on education (% of GDP)	0.07	4.86	4.36	4.79	4.64
Generalized trust	<0.05	24.70	20.56	17.76	21.39
Number of people declaring high trust in Parliament	<0.05	6.17	4.49	2.64	4.56
Active participation in NGOs	<0.05	n/a	2.04	1.48	1.62
Number of people living below $1 per day	0.07	2.84	3.64	1.48	2.82
Schooling indicator: higher education (gross)	<0.05	26.89	41.50	56.05	40.97
Schooling indicator: secondary education (net)	<0.05	80.05	85.12	87.16	85.98
Gini index	<0.05	28.77	33.28	33.37	32.36
Systemic factors					
Health expenditure (% of GDP)	<0.05	6.52	6.68	7.31	6.92
Health expenditure per capita (dol, current)	<0.05	175.24	234.41	632.52	391.38
Public financing on health (% of total expenditures)	0.13	66.62	63.93	63.22	63.96
Out-of-pocket payments (% of total private expenditures)	<0.05	90.99	89.53	88.13	89.12
Spending on hospital care (% of total health expenditures)	<0.05	54.13	41.53	34.80	40.83
Public spending on hospital care (% of total spending on hospital care)	<0.05	95.71	88.02	82.25	85.86
Expenditures on pharmaceuticals (% of total health expenditures)	<0.05	14.42	22.02	26.16	21.88
Public spending on pharmaceuticals (% of total spending on pharmaceuticals)	<0.05	59.92	49,78	44,82	49,16
Practicing physicians/100,000 population	0.70	282,17.	286.15	288.75	285.87
General practitioners/100,000 population	<0.05	31.48	36.94	50.39	42.76
Practicing nurses/100,000 population	<0.05	726.01	644.05	619.74	654.88
Practicing midwifes/100,000 population	<0.05	62.75	42.81	33.84	45.69
Practicing pharmacists/100,000 population	0.08	36.72	33.63	38.62	36.26
Political factors					
No. of political parties in Parliament	0.15	10.79	8.00	11.50	10.18
Seats gained by the political party winning the elections (% of total)	46	43	39	42	42

The comparison of values was completed at the aggregated level, presenting summary characteristics for all post-communist states. To better analyse differences between countries, we constructed a unified synthetic measure of health system outcomes, summing the characteristics (in line with the assigned weights) for each of the countries individually. This enabled us to depict the performance of individual health systems in terms of their outcomes with a single quantitative measure.

The analyses were performed based on one-dimensional (classical or positional descriptive analysis) and two-dimensional (dependency analysis of the characteristics, regression analysis) statistical methods. For estimating the synthetic measure of health system outcomes, we used multidimensional comparative analysis. Zeroed unitarisation was performed to unify the individual variables, which was then followed by a transformation of destimulants and nominants into the stimuli [9, 10]. Since in some cases the data time series were not complete, missing data were supplemented by extrapolation and linear interpolation, and, in special cases, by the mean values. Descriptive statistical analysis of quantitative traits consisted of such measures as the arithmetic mean (xsr), standard deviation (SD) and where needed, the median, among others. A *t*-test compared two average values of normally distributed variables between independent groups, with prior verification of assumptions (the F-test and Levene’s test). To compare two groups with non-normal variable distributions (which applied in most cases), the *U*-test (Mann–Whitney) was used. Comparative analysis of three or more independent groups not normally distributed was performed using the Kruskal-Wallis ANOVA, while tests were conducted with an independent estimation of variance in the case of heterogeneity of variance for variables with normal distributions. The differences between the examined indicators were tested with chi-square tests. The normality of the variables was tested with the Shapiro–Wilk test. To calculate the measure we used an algorithm including the unification and standardisation of variables, which were subsequently weighted and aggregated. For the purpose of standardisation, zeroed unitarisation was used, while aggregation was done in the form of a sum of products of standardised variables and their weights. Calculation of weights was done based on an arbitral assessment of the significance of variables and their dependence on actions by the health system.

**Fig. 1:**
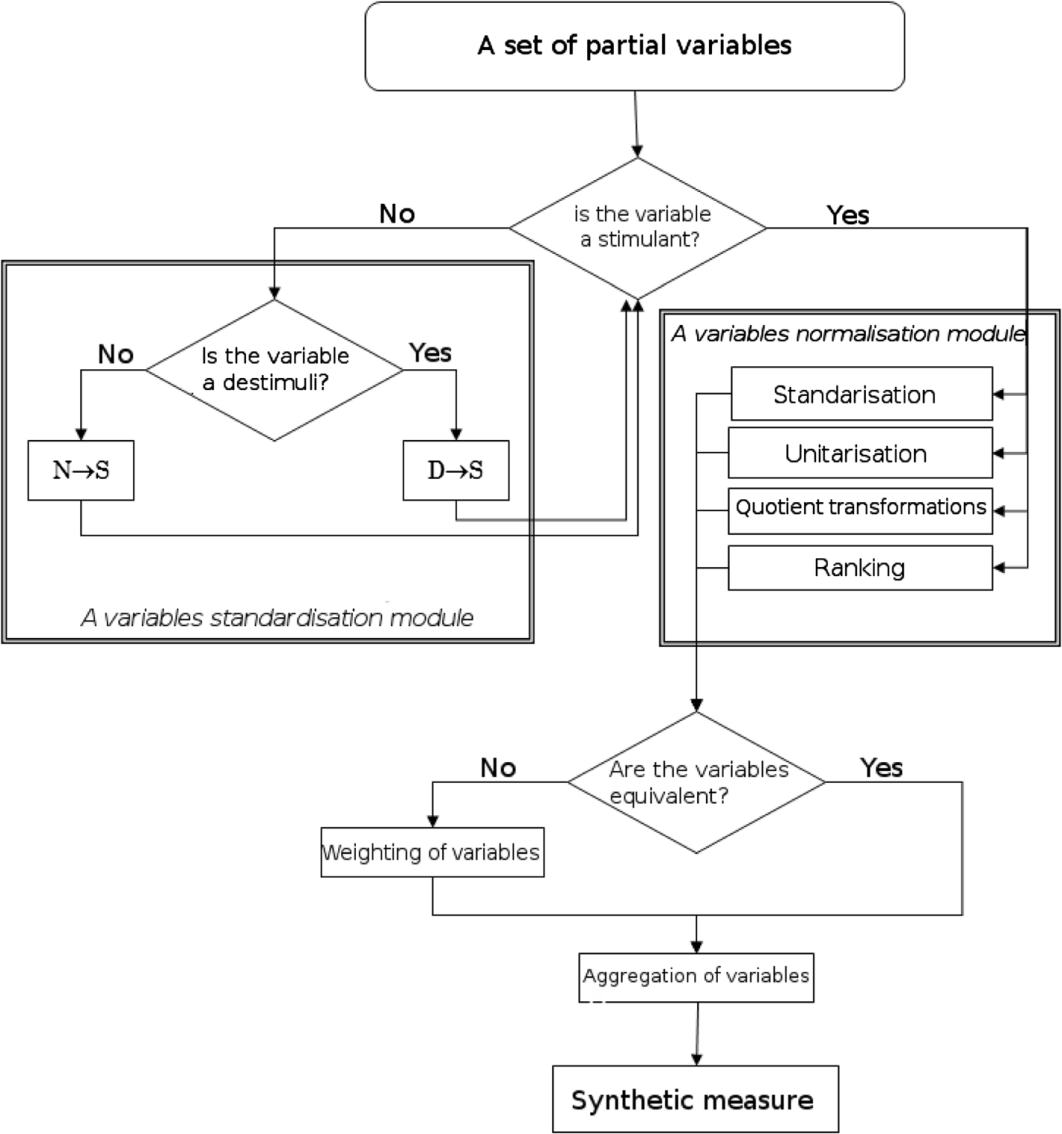
An algorithm of the synthetic measure of health system outcomes construction procedure

The significance level was set at p ≤ 0.05. In correlation analysis, Pearson’s correlation, Spearman’s rho, Kendall tau rank coefficients were used, as were chi-square tests; the appropriate measure depended on the forms of the correlated variables and the distributions of these variables. The synthetic measure of health system outcomes has been constructed in accordance with the algorithm presented on Fig. 1.

The necessary data were retrieved from the World Health Organization (WHO) database in case of the epidemiological data, as well as those relating to health system resources and expenditures. In addition, where necessary, data were also obtained from the databases of the World Bank (most of the macroeconomic data and social factors), and the International Labour Organization (ILO; work accidents, expenditures on social policy). Additional databases used included the World Value Survey and the European Social Survey with regard to indicators on social capital. Data defining the features of the political system were constructed based on the databases of national electoral offices. Since the study did not involved human participants, no ethical consent was required.

## Results

In order to better identify trends in health system outcomes, the entire time series was divided into three sub-periods:

- Period A, covering years 1988–1996;
- Period B, covering years 1997–2004; and
- Period C, covering years 2005–2012.

These periods are largely in line with the reform processes in the CEE countries. Period A corresponds to the first wave of health reforms. Period B was the second wave, partly constituting a reversal or modification of processes initiated during the first wave, and partly being a first wave for some countries. Period C largely corresponded to the period in which the reformed systems are acting in a relatively stable condition.

### The determinants of health system outcomes

Table 1 presents details on potential determinants of changes in health system outcomes during each of the sub-periods, presenting data for all countries combined.

Significant changes between period A and the following periods are noticeable for most of the economic factors. The average GDP per capita in period A was more than 2.5 times lower than in period C. Similar changes were observed in unemployment rates, which increased along with transitional processes, particularly between periods A and B. The economic changes also influenced the structure of the economy, as indicated by the proportions of the GDP generated by industry, agriculture, and services, with a systematic increase in the share of the latter. Interestingly, the industry’s share shrunk very clearly only between periods A and B, while the increase in the share of services in period C appears to correspond only with a decrease in the share of agriculture. Interestingly, the level of spending on social policy declined slightly (and non-significantly), but remained at approximately 17 % of GDP.

In terms of social factors, significant differences are noted for most features, with the exception of public expenditure on education, as well as the percentage of the population living below the poverty line calculated according to country-specific criteria. Similarly, there were no statistically significant differences between periods in the proportion of people living on less than $1 per day, which increased during period B, but reduced markedly in period C.

With regard to the systemic factors, there were no significant differences between the mean levels of healthcare financing from public sources, although they decreased slightly across the periods. Furthermore, there were no significant differences in the numbers of practicing physicians and pharmacists. There has been a significant increase in nominal health expenditure, as well as an increase in total expenditure as a percentage of GDP. In addition, a statistically significant decrease occurred in terms of the share of out-of-pocket funding for private health expenditures. In a very clear manner, the role of the hospital sector in the healthcare system decreased, which was accompanied by an increase in the number of primary care physicians, especially during period C. A constant increase in the financial burden of expenditure on pharmaceuticals was also noticeable. In terms of staffing, a non-significant increase occurred in the number of physicians, accompanied by a decreasing number of nurses and midwives. The overall financial situation of the health systems improved, which was followed by a reduction in patients’ financial burden. Additionally, we observed a systematic reduction in the role of the hospital sector and a shift of responsibility toward primary care. These are likely intentional results of the reforms undertaken, in contrast to the increase in pharmaceutical expenditure.

With regard to political factors, none of the factors analysed showed a statistically significant change. Furthermore, there was no clear trend of changes in this area – the values of the variables changed in different directions.

### Measures of health system outcomes

The picture arising when comparing health system outcomes and the progress in restructuring processes differs from that in the previous analysis (Table 2).

**Table 2:** Features of CEE health systems across sub-periods of transition

Factors		1988–1996	1996–2004	2005–2012	total
	*p*	A	B	C	
Average number of ambulatory visits per person	0.26	7.67	7.09	7.90	7.54
Average number of ambulatory visits per person (change)	<0.05	-0.04	0.01	0.01	-0.01
No. of beds in non-public sector (%)	<0.05	0.02	1.55	8.95	6.38
Bed occupancy	0.01	74.13	69.11	70.00	71.25
Bed occupancy (change)	0.04	-0.02	0.00	0.01	0.00
Average length of stay	<0.05	14.48	11.16	9.05	11.63
Average length of stay (change)	<0.05	-0.01	-0.03	-0.02	-0.02
Hospital beds/100,000 population	<0.05	943.12	715.23	607.44	751.63
Hospital beds/100,000 population (change)	<0.05	0.06	-0.03	-0.01	0.00
Acute beds/100,000 population	<0.05	719.80	532.21	431.42	557.57
Acute beds/100,000 population (change)	<0.05	0.00	-0.03	-0.02	-0.02
Acute beds (% of total hospital beds)	<0.05	0.78	0.76	0.74	0.76
Acute beds as % of total hospital beds (change)	0.52	0.00	0.00	0.00	0.00
Life expectancy	<0.05	70.01	71.60	73.42	71.56
Life expectancy (change)	<0.05	0.00	0.00	0.00	0.00
SDR: circulatory system diseases (0–64 population)	<0.05	153.17	142.15	124.57	141.75
SDR: circulatory system diseases (0–64 population) – change	<0.05	0.01	-0.02	-0.04	-0.01
SDR: circulatory system diseases – total population	<0.05	612.18	597.67	524.34	583.88
SDR: circulatory system diseases – total population (change)	<0.05	0.00	-0.01	-0.03	-0.01
SDR: cancer (change)	<0.05	0.01	0.00	-0.01	0.00
SDR: external causes	<0.05	100.92	92.55	76.42	91.49
SDR: external causes (change)	<0.05	0.02	-0.02	-0.04	-0.01
SDR: tuberculosis	<0.05	18.36	7.09	6.67	11.26
SDR: tuberculosis (change)	<0.05	0.04	-0.03	-0.06	-0.01
SDR: infectious and parasitic diseases	<0.05	21.72	10.75	10.42	14.89
SDR: infectious and parasitic diseases (change)	0.21	0.01	-0.01	0.00	0.00
SDR: diabetes	0.34	24.04	14.92	13.75	18.12
SDR: diabetes (change)	<0.05	0.05	0.02	-0.01	0.02
Infant mortality rate	<0.05	20.05	13.85	9.45	14.89
Infant mortality rate (change)	0.18	-0.04	-0.05	-0.05	-0.05
Maternal mortality	<0.05	36.55	26.40	19.38	27.74
Alcohol consumption per capita	<0.05	8.98	9.11	10.68	9.422
Alcohol consumption per capita (change)	0.18	0.02	0.03	0.02	0.02
No. of daily smokers (% of adult population)	<0.05	31.18	27.90	26.18	28.00
No. of daily smokers (% of adult population) – change	0.57	-0.01	-0.01	-0.01	-0.01
Non-fatal work accidents	0.1368	73540.81	31017.32	23416.62	41785.05
Non-fatal work accidents (change)	0.21	-0.07	-0.03	-0.03	-0.04
Work accidents per 1,000 employees	<0.05	5.23	7.45	5.45	6.42
Work accidents per 1,000 employees (change)	0.61	-0.07	-0.02	-0.04	-0.03
Fatal work accidents (% of all work accidents)	<0.05	39.40	24.95	19.20	27.36
Fatal work accidents (% of all work accidents) – change	0.40	-0.01	-0.02	0.01	-0.01

*SDR* standardized death rate

We noted few significant differences between indicators relating to the use of health services. These values were low in the initial period, after which they slightly increased. The rate of change in the number of outpatient visits appears to be statistically significant. More important statistical results are noticeable in the restructuring of the hospital sector, with the number of hospital beds, both total and acute, decreasing. There was also a statistically significant increase in the number of hospital beds owned by the non-public sector, which occurred both between periods A and B, and B and C. 

In terms of population health outcomes, the observed changes in most cases were significant, both when comparing nominal values and changes over time. We observed non-significant dynamics of change in mortality caused by infectious diseases and parasites and by diabetes (a large decrease can be particularly observed between periods A and B).

Results with regard to health behaviours were more ambiguous. While the number of daily smokers decreased, but with no significant dynamics, alcohol consumption increased. A positive trend is observable in terms of of fatal work accidents, although the dynamics here are not significant if compared between periods.

### Aggregated measure of health system outcomes: country rankings

So far, we presented the general picture of changes in health system outcomes in post-communist countries. In order to analyse the differences between countries, we decided to aggregate the individual measures for each country and construct a unified synthetic measure describing these health system outcomes with a single numerical value. The results are presented in Table 3, averaged for the respective periods A, B, and C. The values assigned to individual countries represent the average value of the synthetic outcome measure for all years in a given time interval. It must be noted that the values themselves do not have any interpretative importance, as they are a result of the adopted aggregation and weighting procedures. The interpretation of the measure values is relevant only in comparative terms between individual countries and periods.

**Table 3:** A synthetic measure of health system outcomes in CEE countries: country rankings

Country	1988–1996 (A)	Rank (A)	1997–2004 (B)	Rank (B)	2005–2012 (C)	Rank (C)	Change A–C	Rank: change A–C
Slovenia	0.79	1	0.82	1	0.85	1	0.07	11
Albania	0.77	2	0.8	4	0.74	15	-0.02	19
Czech Rep.	0.74	3	0.8	2	0.85	2	0.11	5
Slovakia	0.75	4	0.8	3	0.83	5	0.08	8
Macedonia	0.75	5	0.79	6	0.81	8	0.06	14
Croatia	0.75	7	0.8	5	0.84	4	0.09	6
Belarus	0.74	6	0.71	15	0.75	14	0.01	17
Poland	0.73	8	0.78	7	0.84	3	0.11	4
Bulgaria	0.72	9	0.74	12	0.79	9	0.06	12
Romania	0.71	10	0.73	13	0.78	11	0.07	9
Georgia	0.71	11	0.75	11	0.74	16	0.04	16
Hungary	0.7	12	0.77	8	0.82	7	0.11	3
Lithuania	0.7	13	0.76	9	0.77	12	0.07	10
Estonia	0.68	14	0.76	10	0.82	6	0.14	2
Armenia	0.67	15	0.68	16	0.75	13	0.09	7
Ukraine	0.66	16	0.66	17	0.66	19	0	18
Latvia	0.64	17	0.71	14	0.78	10	0.14	1
Moldova	0.63	18	0.63	18	0.69	17	0.06	13
Russia	0.62	19	0.6	19	0.68	18	0.05	15

The range of values of the synthetic measure for period A is 0.162 points, with Slovenia being in first place with a score of 0.7868, and Russia in last place at 0.6248. In this period, Slovenia, Albania, the Czech Republic, Slovakia, Macedonia, Croatia, and Belarus belonged to the group of countries with the highest aggregated level of health system outcomes. The intermediate group included countries such as Poland, Bulgaria, Romania, Georgia, Hungary, Lithuania, and Estonia, while Armenia, Ukraine, Latvia, Moldova, and Russia were among the weakest performing countries in this period.

In period B, differences between countries increased, reaching 0.2289 points. Slovenia and Russia, respectively, remained the best and the weakest countries. Interestingly, in contrast to period A, where the distribution of countries on the scale was quite regular, in period B as many as 11 of the 19 examined countries (Slovenia, the Czech Republic, Slovakia, Albania, Croatia, Macedonia, Poland, Hungary, Lithuania, Estonia, and Georgia) were within the first 30 % of the scale. The intermediate group in this period consisted of Bulgaria, Romania, Latvia, Belarus, and Armenia; the weakest countries in period B were Ukraine, Moldova, and Russia. We can conclude that while the distance between the weakest countries and the best ones increased, the relative differences between the majority of countries decreased. The order of the countries in the ranking showed relatively little change, with a major reshuffling taking place in the middle of the scale. Attention should be drawn to a very significant decrease in the position of Belarus (down from sixth to fifteenth place) and relatively large rises for Hungary, Lithuania, and Estonia.

In period C, we observed a decrease in the difference between the best and the weakest countries in comparison to period B, amounting to 0.1894 points. Unlike in period B, the distribution of countries on the scale becomes more regular, and quite a significant shift of countries on the scale appeared. The countries with the best health system outcomes were Slovenia, the Czech Republic, Poland, Croatia, Slovakia, Estonia, Hungary, and Macedonia. The intermediate group included Bulgaria, Latvia, Romania, Lithuania, Armenia, Belarus, Albania, and Georgia. The weakest performing countries were Moldova, Russia, and Ukraine.

It is worth noting that in each of the three periods under consideration, the group of countries with the weakest outcomes included Moldova, Russia, and Ukraine. In subsequent periods, Armenia and Latvia managed to make progress and leave this group. However, while Armenia remained in the lower parts of the scale across all three periods, there was a significant improvement observable in Latvia, which managed to move to the middle of the ranking by period C. It is also worth noting that Estonia and Poland managed to move to the forefront of the scale. Another country whose ranking changed significantly was Hungary. While in Poland the improvement was mainly observed between periods B and C, in Hungary it took place between periods A and B. On the opposite side was Albania, which showed a drastic decrease in the ranking, especially between periods B and C. Slightly less dynamic, but still significant decreases in the ranking were recorded for Belarus and Georgia.

As an extension of the above considerations, the rate of change in health system outcomes, expressed by the difference in values for the initial and final period of transformation, was analysed. The highest increase was observed in Latvia. High values were also noted for Estonia, Hungary, Poland, the Czech Republic, and Croatia. The worst results – very clearly outlying from other countries – were observed in Belarus, Ukraine, and Albania. The last of these countries is worth special mention, as it was the only country with a negative direction of change.

### Factors correlated with the synthetic measure of health system outcomes

The final stage of the analysis was to identify factors that have a particularly strong correlation with health system outcomes. We observed that almost half of the variables showed significant linear relationships (*p* < 0.05), but most of these relationships were weak.

The details indicated that there was a strong positive correlation between the health system outcome measure and the value of the GDP per capita (*r* = 0.6364). A similar pattern was found for the share of services of total GDP (*r* = 0.4209), while there was an inverse correlation (*r* = -0.3713) with the share of agriculture as a percentage of GDP. However, in both cases, the correlation was weaker than in the case of GDP per capita.

A strong inverse relationship, on the other hand, was found between health system outcomes and the number of people living below the poverty line (*r* = -0.5050). The higher the share of very poor people, the worse were the health system outcomes. Even stronger was the linear correlation between health system outcomes and the Human Development Index (*r* = 0.6925).

Another strong (*r* = -0.7411) negative relationship was found between health system outcomes and the share of spending on hospital services as a percentage of total health expenditure. Obviously, lower spending on hospital services does not mean decreased healthcare financing, but rather a different distribution of funds within the system. Similar correlations were observed between health system outcomes and average length of hospital stay (*r* = -0.7046) and number of beds per 10,000 population (*r* = -0.7352). There was a correlation between health system outcomes and the total number of hospital beds, but it was weaker (*r* = -0.5987) than the former three. It is important to highlight that these factors were also among the components of the aggregate health system outcome measure. However, the strength of the observed correlations, the relatively low weight assigned to these components, and the observed strong correlations suggest that the reduction of the role of the hospital sector had a positive impact on health system outcomes in general.

Correlations between the synthetic health system outcome measure and other relevant factors are presented in Table 4.

**Table 4:** Correlations between the synthetic measure of health system outcomes and other factors

Variable	Correlation coefficient
Economic growth	0.16
GDP per capita	0.64
Unemployment	0.16
Industry share of GDP	-0.17
Services share of GDP	0.42
Agriculture share of GDP	-0.37
Budget deficit	-0.32
Public debt	-0.16
Inflation rate	-0.17
Investments	0.07
Foreign investments	0.03
Foreign investments per capita	0.27
Fiscal burden	-0.17
Public spending on social policy	0.29
People living below poverty line (national threshold)	-0.50
People living for less than $1/day	-0.28
Human Development Index	0.69
Public spending on education	-0.10
Generalized trust	-0.33
Trust in politicians	0.17
Satisfaction with government	0.19
Civic activity	-0.10
Gini index	-0.11
Total health expenditure as a percentage of GDP	0.13
Total health expenditure per capita	0.64
The share of public sources in total expenditure on health	0.32
The share of spending on hospital sector in total health expenditure	-0.74
The share of spending on pharmaceuticals in total health expenditure	0.36
Public spending on pharmaceuticals as a share of total spending on pharmaceuticals	0.43
Expenditure on public health programmes as a percentage of total health expenditure	-0.08
Practicing physicians/100,000 population	-0.37
General practitioners/100,000 population	0.34
Practicing nurses/100,000 population	-0.37
Practicing midwives/100,000 population	-0.38
Practicing pharmacists/100,000 population	0.46
No. of political parties in Parliament	-0.07
Electoral turnout (parliamentary elections)	0.03
No. of seats gained by the election’s winning party (%)	-0.04
No. of government changes	-0.32
No. of beds in non-public sector (%)	0.06
Hospital beds/100,000 population	-0.60
Acute beds/100,000 population	-0.74

## Discussion

The observed differences between countries in terms of health system outcomes allow a number of important insights. First, in most cases, a good initial situation of a country tended to positively determine health system outcomes at later periods. Similarly, countries that entered the transition period at a disadvantage were unlikely to achieve a favourable position in the ranking in subsequent periods.

Second, however, a better starting position did not affect the dynamics of change in subsequent periods, and the differences in health system outcomes between countries are quite significant. A noticeable fact in this regard is the relatively weak performance of countries that emerged from the collapse of Yugoslavia. Although relatively high in the rankings across the periods, these countries did not achieve major improvements, with the exception of Croatia. Interesting observations can also be made when comparing the Baltic states. While major improvements were made by Latvia and Estonia, in Lithuania health system outcomes tended to stagnate. Among the Visegrad Group countries (the Czech Republic, Hungary, Poland and Slovakia), in turn, Slovakia tends to fall short of its neighbours. Finally, the countries emerging from the former Soviet Union (with the exception of the Baltic states) did not achieve major improvements in terms of health system outcomes. All countries in this subgroup are at the lowest parts of the scale – both in the initial transition period and at the end of examined time span. A particularly negative example is Belarus, which had a relatively good starting position, but did not achieve major improvements. On the other hand, Armenia managed to make noticeable progress, although it still belongs to the group with the lowest values of health system outcomes.

Third, countries that did not introduce comprehensive reforms of health financing and organization early on tended to do worse in improving health system outcomes. This applies to Ukraine, Georgia, Belarus. On the other hand, those countries that embarked early on comprehensive reforms tended to do better in improving health system outcomes, although the relationship is neither profound, nor linear. Countries that reformed the earliest, namely during the first half of the 1990s (Hungary, the Czech Republic, Slovakia, Estonia, Latvia, and Croatia) experienced a higher growth in health system outcomes between periods. At the same time, there are exceptions to this rule, such as Russia, Albania, and Slovenia. On the other hand, among countries where the reform process was more spread out over time or started later, only Poland managed to make significant improvements in health system outcomes.

The analysis of social factors revealed interesting features of post-communist countries. While the measures defining the standard of living (Human Development Index [HDI], schooling factors, percentage of population living in poverty) systematically improved with economic development, there were simultaneous decreases in factors that define civic activity and social capital, such as involvement in NGOs, generalized trust, or confidence in political institutions. This means that in CEE countries the transition generally improved the quality of life, but this was perhaps accompanied by feelings of instability, fatigue with the progressing transition, and the conviction that the transition increased economic inequalities (as seen in the Gini index). As some previous studies show, these trends negatively influence the level of social capital [11, 12].

There is no literature that would allow for a full and meaningful comparison of our results with other similar studies. Although the literature on post-communist countries is quite extensive, most of the existing studies focus only on selected aspects of the functioning of the health system or are purely descriptive. There is also a lack of studies on the effects of implemented reforms. Comparisons are additionally hindered by the multiplicity of applied methodologies.

The results of our study are consistent, among others, with the a study by Adeyi, et al. [13], which noted a positive relationship between GDP growth and health status in post-communist countries. However, the study also highlighted exceptions to this rule, namely countries in which the health status of the population decreased particularly sharply in the initial period of transformation. These countries were Hungary, Bulgaria, Ukraine, and Russia. This finding is consistent with the results of our study which indicated that the same countries were among the lowest achieving ones in terms of health system outcomes in the first decade of transition. Another study addressed the relation between circulatory system diseases and the family and educational status of patients in CEE [14]. Notably, there were only weak relationships between level of education, family status, and the risk of certain cardiovascular diseases. The study suggested that the risk factors were to be found in other places, which is consistent with the findings presented in our study.

A slightly different observation was made by Álvarez-Dardet and Franco-Giraldo [15] with regard to the relationship between the health status of the population and political conditions. While our study did not show any correlation, this previous study showed a link between health status and the deficit of democracy, as defined based on data derived from Freedom House. This may suggest that, while the formal structure of the political system does not significantly affect the functioning of the health system, its actual consistency with adopted standards may significantly affect health system outcomes.

Our study presented data on health system outcomes in CEE countries across the transition period. As such, this study broadens the existing knowledge on the course and results of reform processes. However, it has important limitations. Our study does not provide a clear answer as to which reform elements were most beneficial for health system outcomes. Identifying such components is intrinsically difficult, due to the variety of options and combinations in individual countries. It is even conceivable that the identification of a single path to improve health system outcomes is not possible [1, 16]. It would require an in-depth analysis of the content of reforms in individual countries which was outside the scope of our study. However, our observations are consistent with the findings of other studies that discuss health reforms in post-communist countries. According to these previous studies, factors crucial to improved health system outcomes include a general financial stability of the health system, as well as a reasonable distribution of the financial burden of health expenditure between the patient and the public sector that provides financial protection against catastrophic expenditure. Our study also confirmed the previous finding that better results in terms of health system strengthening tended to be achieved by countries deciding to embark on comprehensive health reforms early on [17]. However, in our analysis, while a better initial situation determined the actual values of the outcome measure across subsequent periods, it did not appear to positively influence subsequent improvements. Indeed, countries that had a better starting position often failed to make major improvements in the period of transition. Relatively good starting positions at the end of the communist period explain the relatively high position occupied by the former Yugoslav republics, particularly Macedonia, which managed to maintain a good level of health system outcomes because of the legacy of the previous system [16]. However, these countries were not able to make significant improvements in the period of transition.

## Conclusions and recommendations

1. CEE countries, when analysed in total, managed to achieve significant improvements in measures defining the health status of their populations over the transition period. There were also significant changes in the structure and resources of the health system, although the trends are not as unambiguous as with regard to health outcomes. Changes in individual measures were often not statistically significant. This was observed in the number of total and acute hospital beds, infant mortality, consumption of alcohol and tobacco, and fatal accidents at work.
2. The best performing health systems were Slovenia, the Czech Republic, Poland, Croatia, Slovakia, Estonia, Hungary, and Macedonia, while the weakest were Moldova, Russia, and Ukraine. If we consider only the progress in health system outcomes during the transition period, the greatest improvement was achieved by Latvia, followed by Estonia, Hungary, Poland, the Czech Republic, and Croatia. The least improvements were achieved by Belarus, Ukraine, and Albania. When comparing the effects and dynamics of change in each of the countries, a better initial position in most cases positively determined health system outcomes at later stages, although it did not affect the degree of improvements. In addition, countries that embarked on comprehensive reforms early on tended to achieve the greatest improvements in health system outcomes.
3. The trends in health financing were consistent with the changes in health system outcomes. Among the factors that seemed to positively stimulate improvements in health system outcomes were the total expenditure on health and a lower financial burden on patients. We could not find correlations between health system outcomes and factors relating to the social sphere or formal political life.

Overall, it seems that health system outcomes were determined primarily by the broader economic context of the country in question. The aggregated outcome measure showed a much higher correlation with absolute GDP than with the level of health care expenditure as a percentage of GDP. This suggests that there are limits to how far poorer countries can improve health system outcomes by committing more financial resources to the health system. At the same time, however, increasing the share of public financing for health is beneficial for improving health system outcomes. There is clear scope for further improvements in this area, in line with increasing calls for universal health coverage.

Another area where much more progress can be made relates to health behaviours, such as the consumption of alcohol or tobacco. Our analysis suggests that policies to address these health behaviours have so far been insufficient or ineffective. Finally, more studies are needed that explore the effects of health reforms in post-communist countries and identify best practices in strengthening health systems and improving health system outcomes.

### Limitations of the study and applied methodology

The aim of the study was to investigate the evolution of the health systems outcomes in CEE countries. Based on the results it is possible to draw a general conclusions relating to the effectiveness of health reforms implemented in individual country. Nonetheless, this study does not constitute an analysis of the effectiveness of specific reform solutions, although it may provide a basis for taking such studies. In such a case it is important to underline the fact that the health status of the population is determined by a number of factors, not just reforms carried out in the health system, which is also reflected in our study.

In terms of the applied methodology there is also a set of limitations that is needed to be outlined. First, the data sources used were not always fully complete, which necessitated the use of compensatory mechanisms. Nonetheless, the observed regularity in trends of indicator value changes, as well as the relatively small scale of the identified gaps, allows to assume that the decline in the precision of the result should be considered as small and does not significantly affect its credibility.

Secondly – to maximise the objectivity of the result, we based our calucations on data available in the databases of globally active international organizations. However, there is no full certainty that the data for individual countries are free of interference related to the potential lack of precision in reporting or estimating the value of individual epidemiological or systemic indicators. The differences appearing in the data when obtained from different sources confirm this possibility. At the same time, however, this problem might affect the specific values of the calculated synthetic health system outcome measure, but due to the complexity of the measure, as well as wide range of data used, interference should not cause a significant decrease in the reliability of analysis relating to the global trends in individual countries and comparisons between them. Even in case of the lack of full precision in mapping the reality, the total credibility of the result should be viewed as sufficient.

Thirdly, the methodology used for estimating the synthetic outcome measure in terms of the selection of partial variables and their weghts to some extent is arbitral and as such may be a subject to discussion. Again, it should be noted however that the modification of measure used in the above ranges may affect the numerical values characterising individual countries, but its comparative and analytical value basically should remain unchanged or change in a small scale only.

## Abbreviations

CEE: Central and Eastern Europe
GDP: gross domestic product
HDI: Human Development Index
ILO: International Labour Organisation
NGO: Non-governmental Organisation
WHO: World Health Organisation
